# Isolation of Mesenchymal Stem Cells from Human Deciduous Teeth Pulp

**DOI:** 10.1155/2017/2851906

**Published:** 2017-03-09

**Authors:** Aileen I. Tsai, Hsiang-Hsi Hong, Wey-Ran Lin, Jen-Fen Fu, Chih-Chun Chang, I-Kuan Wang, Wen-Hung Huang, Cheng-Hao Weng, Ching-Wei Hsu, Tzung-Hai Yen

**Affiliations:** ^1^Department of Pediatric Dentistry, Chang Gung Memorial Hospital and College of Medicine, Chang Gung University, Linkou, Taiwan; ^2^Department of Periodontics, Chang Gung Memorial Hospital and College of Medicine, Chang Gung University, Linkou, Taiwan; ^3^Department of Gastroenterology and Hepatology, Chang Gung Memorial Hospital and College of Medicine, Chang Gung University, Linkou, Taiwan; ^4^Department of Medical Research, Chang Gung Memorial Hospital and College of Medicine, Chang Gung University, Linkou, Taiwan; ^5^Department of Clinical Pathology, Far Eastern Memorial Hospital, New Taipei, Taiwan; ^6^Department of Nephrology, China Medical University Hospital and College of Medicine, China Medical University, Taichung, Taiwan; ^7^Poison Center, Department of Nephrology, Chang Gung Memorial Hospital and College of Medicine, Chang Gung University, Linkou, Taiwan; ^8^Kidney Research Center, Chang Gung Memorial Hospital, Linkou, Taiwan; ^9^Center for Tissue Engineering, Chang Gung Memorial Hospital, Linkou, Taiwan

## Abstract

This study aimed to identify predictors of success rate of mesenchymal stem cell (MSC) isolation from human deciduous teeth pulp. A total of 161 deciduous teeth were extracted at the dental clinic of Chang Gung Memorial Hospital. The MSCs were isolated from dental pulps using a standard protocol. In total, 128 colonies of MSCs were obtained and the success rate was 79.5%. Compared to teeth not yielding MSCs successfully, those successfully yielding MSCs were found to have less severe dental caries (no/mild-to-moderate/severe: 63.3/24.2/12.5% versus 12.5/42.4/42.4%, *P* < 0.001) and less frequent pulpitis (no/yes: 95.3/4.7% versus 51.5/48.5%, *P* < 0.001). In a multivariate regression model, it was confirmed that the absence of dental caries (OR = 4.741, 95% CI = 1.564–14.371, *P* = 0.006) and pulpitis (OR = 9.111, 95% CI = 2.921–28.420, *P* < 0.001) was significant determinants of the successful procurement of MSCs. MSCs derived from pulps with pulpitis expressed longer colony doubling time than pulps without pulpitis. Furthermore, there were higher expressions of proinflammatory cytokines, interleukin- (IL-) 6 and monocyte chemoattractant protein- (MCP-) 1, *P* < 0.01, and innate immune response [toll-like receptor 1 (TLR1) and TLR8, *P* < 0.05; TLR2, TLR3, and TLR6, *P* < 0.01] in the inflamed than noninflamed pulps. Therefore, a carious deciduous tooth or tooth with pulpitis was relatively unsuitable for MSC processing and isolation.

## 1. Introduction

The presence of stem cells in the dental pulp of a deciduous tooth [1] is an exciting new finding that has significant meaning for the pediatric dental practitioners. When compared to other types of adult stem cells [2], obtaining stem cells from teeth is ethically noncontroversial, noninvasive, less dependent on timing, and far less expensive. Unlike embryonic cells, which are extracted from human embryos, generating stem cells from dental pulp is a relatively noninvasive and noncontroversial process.

Every child loses deciduous teeth, which creates the perfect opportunity to recover and store this source of stem cells. Stem cells from teeth replicate at a faster rate and for a longer period of time than do stem cells harvested from other tissues of the body [3]. Another advantage of dental stem cells is that there is more than one opportunity to harvest teeth stem cells from deciduous teeth. The problem is that since pulpitis can be caused by extensive dental caries, it is unknown whether the presence of caries or pulpitis could affect the harvesting of MSCs from deciduous teeth. Our previous survey [4] also indicated a high level of untreated dental caries (56%) among children less than 6 years of age. Another untapped question is as follows: are stem cells collected more easily from a supernumerary than normal deciduous tooth?

Several studies have investigated whether dental pulp stem cells (DPSCs) isolated from teeth with pulpitis can survive and retain their tissue regenerative potential. Alongi et al. [5] showed that inflamed dental pulps expressed higher levels of stromal cell-derived factor-1 (STRO-1), CD90, CD105, and CD146 compared to normal pulps. However, the doubling of the total population was lower for DPSCs isolated from inflamed pulps than for DPSCs isolated from normal pulp [5]. Wang et al. [6] reported that putative stem cells isolated from teeth with irreversible pulpitis showed decreased ability for colony formation and a slightly decreased cell proliferation rate but had similar levels of STRO-1 expression and exhibited a similar percentage of positive ex vivo osteogenic induction [6]. Jiang et al. [7] reported an enhanced expression of the STRO-1/CXC chemokine receptor 4 axis in inflamed dental pulp tissues. Pereira et al. [8] demonstrated that the morphology, proliferation rate, and differentiation potential of DPSC from inflamed pulps were similar to the observed in normal pulps. Yu et al. [9] revealed that mRNA of inflammatory factors, including IL-1 beta, IL-6, and tumor necrosis factor- (TNF-) alpha, was expressed at similar levels in both stem cells from inflamed pulp of deciduous teeth and stem cells from exfoliated deciduous teeth, but the stem cells from inflamed pulps of deciduous teeth secreted more TNF-alpha protein. Interestingly, Kim et al. [10] disclosed that fibroblastic growth factor-2 applied to stem cells from inflamed pulp tissue of deciduous teeth during expansion enhanced the colony-forming efficiency of these cells, increased their proliferation and migration potential, and reduced their differentiation potential in vitro. Finally, Lee et al. [11] presented that DPSC isolated from either inflamed or noninflamed pulps possessed stem cell properties and suppress macrophage functions via the TNF-alpha/indoleamine-pyrrole 2,3-dioxygenase axis.

On the other hand, the objective of this study was to identify predictors of success rate of MSCs isolation from these deciduous teeth pulp. It was an intriguing question with clinical implications, and that was how the condition of the tooth and/or pulp affected the ability to isolate MSC populations.

## 2. Materials and Methods

The study protocol complied with the guidelines of the Declaration of Helsinki and was approved by the Medical Ethics Committee of Chang Gung Memorial Hospital, a tertiary referral center located in the northern part of Taiwan. In addition, informed written consent for tooth extraction and MSC processing and isolation was obtained from the parents of all the children before the procedures were performed.

### 2.1. Clinical Data

A total of 161 deciduous teeth were extracted at the pediatric dental clinic of Chang Gung Memorial Hospital. A complete clinical profile of each patient was recorded using a standardized form. Data were obtained for each patient regarding the following parameters: age, sex, type of teeth, underlying diseases, interval between extraction to culture, severity of dental caries, and presence or absence of pulpitis.

### 2.2. Primary Cell Culture

Deciduous teeth were extracted and transported to the laboratory in Dulbecco's phosphate buffered saline (DPBS; Gibco, Invitrogen, Carlsbad, CA) solution containing 300 unit/mL penicillin and 300 *μ*g/mL streptomycin (Gibco) and maintained at 4°C [12]. After beta-iodine sterilization, the dental pulp tissue was separated from the pulp chamber and root canal, which was revealed by cutting around the cementoenamel junction by using sterilized dental burs. After separation, the dental pulp was isolated using a barbed broach or a sharp excavator. The dental pulp was then minced and cultured at 5% CO_2_ atmosphere under 37°C in a 35 mm culture disk containing passage 0 medium with *α*-modified Eagle's medium (Hyclone, Logan, UT, USA), 10% fetal bovine serum (Hyclone), 300 unit/mL penicillin, and 300 *μ*g/mL streptomycin (Gibco). The medium was changed after 3 days and, thereafter, 2 times per week. By approximately 7 days, small colonies had formed. The passage 1 medium contained alpha-modified Eagle's medium (Hyclone), 10% fetal bovine serum (Hyclone), 100 unit/mL penicillin, and 100 *μ*g/mL streptomycin (Gibco). Each subsequent passage was performed after 70% confluence was achieved.

### 2.3. Flow Cytometry

The passage 3 cells were cultured in a T25 flask until they reached full confluence. They were then washed twice with PBS and subsequently treated with 0.25% trypsin (25200; Gibco) at 37°C. Trypsin reaction was stopped by the addition of the medium, and the cell suspension was transferred to a polystyrene round-bottom test tube (352054; Falcon, BD Labware, Franklin Lakes, NJ). The suspension was centrifuged at 1500 rpm for 5 min. The supernatant was then discarded, and the cell pellet was washed twice with PBS. After being treated with permeabilization buffer (00-8333; eBioscience, San Diego, CA, USA), the cells were incubated with the primary antibody at 4°C for 30 min. The results were analyzed using the FACSCalibur™ system (BD Bioscience, San Jose, CA, USA). The primary antibodies used were CD73 (PE, 550257; BD Pharmingen), CD90 (APC, 559869; BD Pharmingen), STRO-1 (Alexa Fluor 647; BioLegend, San Diego, CA), CD44 (555479; BD Pharmingen), CD45 (FITC, 555482; BD Pharmingen), CD34 (FITC, 130081001; Miltenyi Biotec, Auburn, CA), CD19 (FITC, 555412; BD Pharmingen), and HLA-DR (FITC, 555811; BD Pharmingen).

### 2.4. Colony-Forming Units

The passage 0 generation cells were grown in media until 90% confluence was achieved and reseeded at 10 × 10^3^, 5 × 10^3^, and 1 × 10^3^ cells into new culture disks and incubated for 7–14 days. When the cell colonies grew to include approximately 50 cells, the media were removed, washed twice with PBS, and fixed with 10% formaldehyde (H121-08; Mallinckrodt Chemicals, Phillipsburg) for 30 min. After washing twice with PBS, the cells were stained with 0.1% crystal violet (C3886; Sigma-Aldrich) for 15 min and then washed 3–5 times with double distilled water. The dishes were subsequently photographed to determine the number of colony-forming units.

### 2.5. Colony Doubling Time

For growth rate analysis, cells were plated in a culture disk at a concentration of 1 × 104 cells. After reaching 80–90% confluence (around 5–7 days), cells were collected, washed with PBS, and treated with 0.25% trypsin (25200, Gibco, Invitrogen, Carlsbad, CA) at 37°C. Then, stopped trypsin reaction by adding medium and transferred the cell suspension to an Eppendorf Tube. The suspension was centrifuged (1500 rpm for 5 minutes), supernatant discarded, and cell pellet was washed twice with PBS. The number of cells was then counted using a hemocytometer. Then, 1 × 104 cells were reseeded into another culture disk to obtain next generation of cells. After reaching 80–90% confluence (around 5–7 days), the number of cells was counted again. The doubling time was calculated using online formula (web address: http://www.doubling-time.com/compute.php).

### 2.6. Differentiation Study

For osteoblastic differentiation, passage 3 cells were plated in a culture disk at a concentration of 1 × 10^4^ cells. After reaching 70–80% confluence, the cells were treated with osteoblastic differentiation medium containing alpha-modified Eagle's medium (Hyclone), 10% fetal bovine serum (Hyclone), 10 mM beta-glycerophosphate (G-6376; Sigma-Aldrich), 10^−7 ^M dexamethasone (D4902; Sigma-Aldrich), and 100 *μ*M ascorbic acid 2-phosphate (Sigma-Aldrich). The cells were cultured for 4–6 weeks. Osteoblastic differentiation was verified by von Kossa staining. The procedure involved washing the cells twice with PBS and fixing them for 30 min with 10% formaldehyde (H121-08; Mallinckrodt Chemicals). After washing twice with double distilled water, the sample was incubated with 5% silver nitrate solution (S0139; Sigma-Aldrich) and then placed under ultraviolet light for 60 min. This was followed by washing the cells 3 times with 5% sodium thiosulfate (S7026; Sigma-Aldrich) and 3 times with double distilled water. Mineralization was assessed by microscopic examination.

For chondrocytic differentiation, the passage 3 cells were plated in a culture disk, at a concentration of 1 × 10^4^ cells. After reaching 70–80% confluence, the cells were cultured for 4–6 weeks in chondrocytic differentiation medium containing *α*-modified Eagle's medium (Hyclone), 10% fetal bovine serum (Hyclone), 10 mM beta-glycerophosphate (G-6376; Sigma-Aldrich), 10^−7 ^M dexamethasone (D4902; Sigma-Aldrich), 100 M ascorbic acid 2-phosphate (Sigma-Aldrich), and 120 g/L transforming growth factor-*β* (240-B; R&D Systems, Minneapolis, MN, USA). Chondrocytic differentiation was determined using Alcian blue staining. The procedure involved washing the cells twice with PBS and fixing them for 30 min with 10% formaldehyde (H121-08; Mallinckrodt Chemicals). After washing twice with double distilled water, the cells were incubated with 3% acetic acid (JT Baker, Phillipsburg, NJ, USA) for 2 min. The cells were then washed 3 times with double distilled water and then incubated in 1% Alcian blue 8GX (in 3% acetic acid solution at pH 2.5) for 30 min. Then, the cells were washed 3 times with double distilled water and observed under a microscope for sulfated proteoglycan deposition.

For adipocytic differentiation, the passage 3 cells were plated in a culture disk at a concentration of 1 × 10^4^ cells. After reaching 70–80% confluence, the cells were cultured for 8–12 weeks in adipocytic differentiation medium containing alpha-modified Eagle's medium (Hyclone), 10% fetal bovine serum (Hyclone), 0.5 mM 3-isobutyl-1-methylxanthine (I5879; Sigma-Aldrich), 10^−6 ^M dexamethasone (D4902; Sigma-Aldrich), 5 *μ*g/mL insulin, and 60 *μ*M indomethacin (I7378; Sigma-Aldrich). Adipocytic differentiation was detected using Oil Red O. The procedure involved washing the cells twice with PBS and fixing them for 30 min in 10% formaldehyde (H121-08; Mallinckrodt Chemicals). After washing twice with double distilled water, the cells were incubated with 0.5% Oil Red O (O-0625; Sigma-Aldrich) for 10 min. Next, the cells were washed 3 times with 60% isopropanol (JT Baker) and 3 times with double distilled water. They were then observed under a microscope for fat deposition.

### 2.7. Real-Time Polymerase Chain Reaction (PCR)

Total ribonucleic acid (RNA) was extracted from frozen kidneys and real-time PCR was performed as described previously [13]. Real-time PCR was performed on an ABI-Prism 7700 using SYBR Green I as a double-stranded DNA-specific dye according to the manufacturer's instructions (PE-Applied Biosystems, Cheshire, UK). The glyceraldehyde-3-phosphate dehydrogenase (GADPH) mRNA expression was simultaneously measured as an internal control. Primers (Table 1) were constructed to be compatible with a single RT-PCR thermal profile (95°C for 10 min, 40 cycles of 95°C for 30 s, and 60°C for 1 min). The number of cycles to the threshold of detecting fluorescence was monitored in real time using an ABI-Prism 7700 (PE-Applied Biosystems). All mRNA expressions were expressed relative to the GAPDH mRNA expression, and the magnitude (in folds) of changes in the gene expressions was determined in comparison with the controls.

### 2.8. Individual Tooth Status and Caries Classification

In this study, the extraction of teeth and recording of tooth status, tooth type, tooth number, and general information of each patient were performed by a pediatric dentist. The severity of dental caries was classified according to the criteria of the International Caries Assessment and Detection System [14]: no caries (tooth sound), mild/early (first visual change in enamel or distinct visual change in enamel), moderate/established (localized enamel breakdown or underlying dentin shadow), and severe (distinct cavity with visible dentin or extensive cavity with visible dentin). In this study, teeth were categorized into 3 levels according to the severity of caries: (1) sound, teeth with no evidence of treated or untreated clinical caries; (2) mild-to-moderate caries, teeth showing obvious cavitation or those that had been restored with dental materials; and (3) severe caries, teeth that were treated with pulpotomy or pulpectomy. Four types of deciduous teeth were collected: incisors, canines, molars, and supernumerary teeth. The pulpal status was classified as (1) no pulpitis: sound or restored teeth and (2) pulpitis: teeth with pulpectomy or pulpotomy. The pulpal treatments were confirmed by clinical examination and dental radiography.

### 2.9. Statistical Analysis

Continuous variables were expressed as means with the standard deviation for the number of observations (mean ± standard deviation), whereas categorical variables were expressed as numbers with the percentages in parenthesis, that is, as *n* (%). All data were routinely tested for normality of distribution and equality of standard deviation before analysis. Comparisons of continuous variables between the successful or unsuccessful MSC isolation groups were performed using Student's *t*-test. Chi-square or Fisher exact test was used for categorical variables. An initial univariate logistic regression analysis was performed to compare the frequency of potential covariates associated with the successful isolation of MSCs. To control the confounding factors, a multivariate logistic regression analysis (stepwise backward approach) was performed to analyze the significant covariates (*P* < 0.10) identified on simple logistic regression. The criterion for significance was a 95% confidence interval (CI) to reject the null hypothesis. Statistical analyses were performed using IBM SPSS Statistics Version 20.

## 3. Results

### 3.1. Isolation and Culture of MSCs

Most of the MSCs were spindle-shaped and fibroblast-like, while some were cuboidal or polygonal in appearance. The MSCs exhibited colony-forming efficiency on crystal violet staining.

### 3.2. In Vitro Differentiations

The differentiation studies demonstrated that the MSCs could be subpassaged and differentiated in vitro into a variety of cells of the mesenchyme lineages, such as osteoblasts (von Kossa staining), chondrocytes (Alcian blue staining), and adipocytes (Oil Red O staining), under an appropriate culture medium.

### 3.3. MSC Surface Markers

Immunophenotype was ascertained by flow cytometry analysis. The analysis showed that MSCs were positive for the expressions of CD73, CD90, STRO-1, and CD44, but negative for the expressions of CD45, CD34, CD19, and HLA-DR.

### 3.4. Baseline Characteristics

In all, 128 colonies of MSCs were obtained from 161 deciduous teeth. The success rate was 79.5% (Table 2). Compared to teeth not yielding MSCs successfully, those successfully yielding MSCs were found to have less severe dental caries (no/mild-to-moderate/severe: 63.3/24.2/12.5% versus 12.5/42.4/42.4%, *P* < 0.001) and less frequent pulpitis (no/yes: 95.3/4.7% versus 51.5/48.5%, *P* < 0.001). No other significant differences were noted in the other clinical variables.

### 3.5. Determinants of Successful Isolation of MSCs

Multivariate logistic regression analysis showed that the absence of dental caries (OR = 4.741, 95% CI = 1.564–14.371, *P* = 0.006) and pulpitis (OR = 9.111, 95% CI = 2.921–28.420, *P* < 0.001) was significant determinants for the successful isolation of MSCs (Table 3). The data suggest that a caries-free tooth or a tooth free of pulpitis had 4.741-fold or 9.111-fold greater chance of yielding a successful harvest of MSCs than a tooth with caries or pulpitis, respectively.

### 3.6. Colony Doubling Time

**Figure 1:**
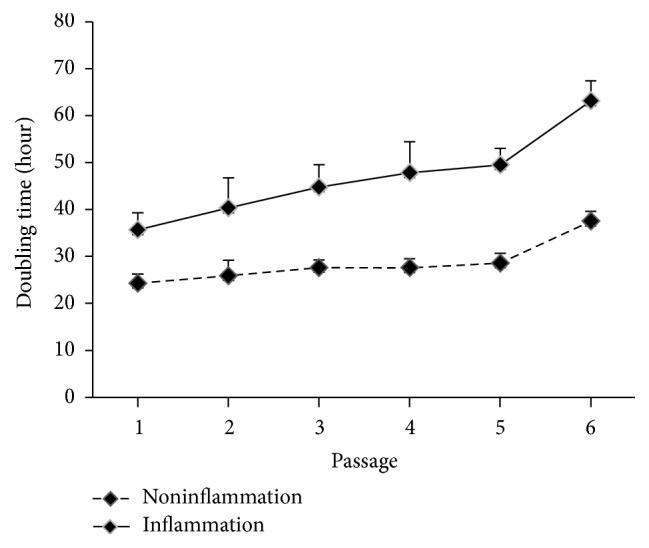
Colony doubling time. MSCs derived from pulps with pulpitis expressed longer colony doubling time than pulps without pulpitis.

It was revealed that MSCs derived from pulps with pulpitis expressed longer colony doubling time (passage 1: 35.6 ± 3.7 versus 24.2 ± 2.0 hours, *P* < 0.001; passage 2: 40.3 ± 6.4 versus 25.9 ± 3.3 hours, *P* = 0.002; passage 3: 44.8 ± 4.8 versus 27.6 ± 1.6 hours, *P* < 0.001; passage 4: 47.8 ± 6.6 versus 25.6 ± 1.9 hours, *P* < 0.001; passage 5: 49.5 ± 3.5 versus 28.6 ± 2.1 hours, *P* < 0.001; passage 6: 63.1 ± 4.3 versus 37.5 ± 2.1 hours, *P* < 0.001, Figure 1) than MSCs derived from pulps without pulpitis.

### 3.7. Proinflammatory Cytokines and Innate Immune Response

**Figure 2:**
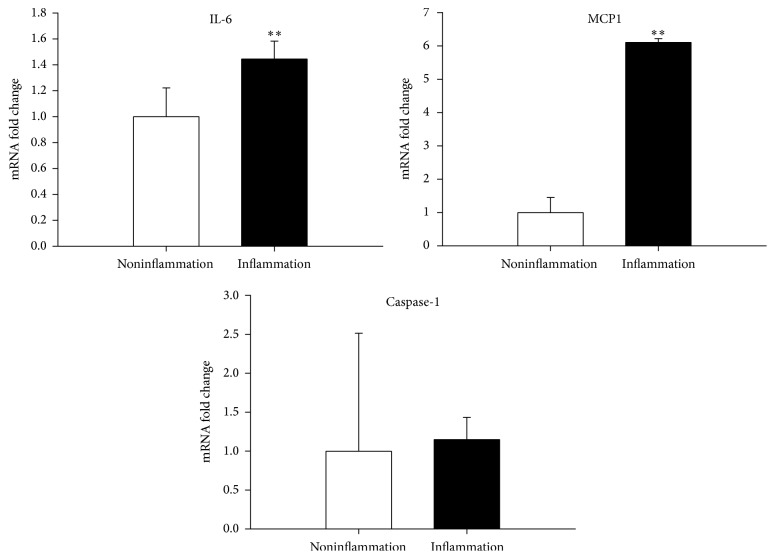
Proinflammatory cytokines. Real-time PCR analysis demonstrated that dental pulps with pulpitis had higher expressions of proinflammatory cytokines (IL-6, *P* < 0.01 and MCP-1, *P* < 0.01) than pulps without pulpitis. ^*∗∗*^*P* < 0.01.

**Figure 3:**
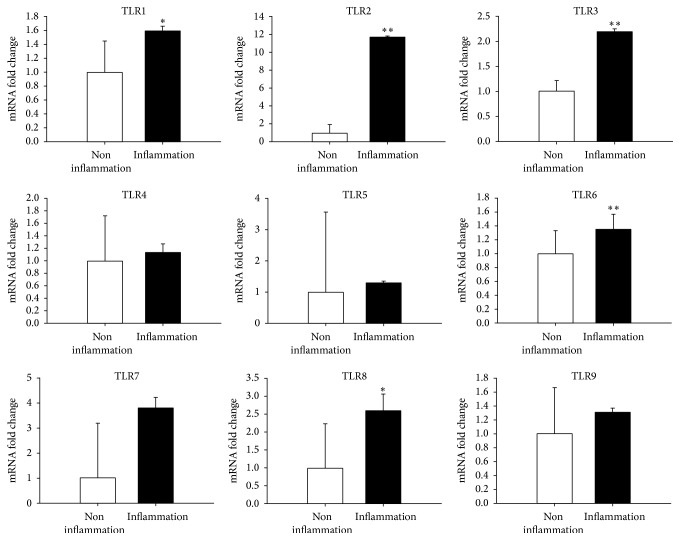
Innate immune response. Real-time PCR analysis revealed that dental pulps with pulpitis had higher expressions of innate immune response (TLR1, *P* < 0.05; TLR2, *P* < 0.01; TLR3, *P* < 0.01; TLR6, *P* < 0.01; TLR8, *P* < 0.05) than pulps without pulpitis. ^*∗*^*P* < 0.05; ^*∗∗*^*P* < 0.01.

It was demonstrated that pulps with pulpitis had higher expressions of proinflammatory cytokines (IL-6, *P* < 0.01 and MCP-1, *P* < 0.01, Figure 2), and innate immune response (TLR1, *P* < 0.05; TLR2, *P* < 0.01; TLR3, *P* < 0.01; TLR6, *P* < 0.01; TLR8, *P* < 0.05, Figure 3) than pulps without pulpitis.

## 4. Discussion

Many adult tissues contain a population of stem cells that have the ability to regenerate after damage [2, 15–18]. Recently, much interest has been generated by dental tissue-derived MSCs and their roles in maintaining the physiological structure of dental tissues [19–21]. Nevertheless, the most important part of this data is that a tooth free of caries or pulpitis had 4.741-fold or 9.111-fold greater chance of successful MSC isolation than those with caries or pulpal disease, respectively. This clinical study, based on a considerable number of human samples (*N* = 161), provided evidence that a carious deciduous tooth or tooth with pulpitis was relatively unsuitable for MSC processing and isolation.

In this study, deciduous teeth were extracted and processed using a standard clinical and laboratory procedure. Cells were isolated from human deciduous dental pulp, STRO-1 sorted, and seeded out to obtain colony-forming units. The cultured cells also fulfilled the minimal criteria for human MSCs [22]. In 2006, the Mesenchymal and Tissue Stem Cell Committee of the International Society for Cellular Therapy [22] proposed a set of criteria to define human MSCs. First, MSCs must adhere to plastic when maintained in standard culture conditions. Second, MSCs must test positive for markers of immaturity, such as CD73, CD90, and CD105, and negative for markers of maturity, such as CD45, CD34, CD14, CD11b, CD79 alpha, CD19, and HLA-DR surface molecules. Third, MSCs must differentiate to osteoblasts, adipocytes, and chondroblasts under standard in vitro differentiating conditions [22]. Notably, a routine in vivo animal transplantation study is not required to characterize human MSCs.

We found that deciduous teeth successfully yielding MSCs had less severe dental caries (*P* < 0.001) and less frequency of pulpitis (*P* < 0.001) than those not yielding MSCs. This can be explained by the fact that untreated dental caries may progress to pulpitis and, eventually, to necrosis of the pulp, with minimal sparing of vital tissues [4]. Recent studies [5–7] have also indicated that although MSCs can be isolated from permanent teeth with pulpitis, but some of the stem cell/regenerative properties were altered and impaired. Our analysis revealed that MSCs derived from pulps with pulpitis expressed longer colony doubling time than pulps without pulpitis (Figure 1). Furthermore, deciduous pulps with pulpitis suffered higher expressions of proinflammatory cytokines (IL-6 and MCP-1) and innate immune response (TLR1, TLR2, TLR3, TLR6, and TLR8) than pulps without pulpitis (Figures 2 and 3). Tomic et al. [23] revealed that treatment of DPSCs with toll-like TLR3 agonist potentiated transforming growth factor-beta and IL-6 secretions by these cells. On the other hand, Tom-Kun Yamagishi et al. [24] confirmed that the suppressing effect of porphyromonas gingivalis lipopolysaccharide on mineralized matrix formation by human DPSCs was moderated by TLR2 blockade. In another study, Liu et al. [25] showed that TLR4 was expressed in the odontoblast layer and areas that colocalized with blood vessels to different levels in healthy teeth and teeth affected by caries. TLR4 mRNA, TLR4 protein, and mRNA of cytokine levels could be elevated with stimulations of lipopolysaccharide and extracts from Streptococcus mutans. The lipopolysaccharide and extracts from* S*.* mutans* treatment inhibited the proliferation of DPSCs but promoted migration. Finally, Fawzy El-Sayed et al. [26] described a distinctive pattern of TLR expression profile of DPSCs in uninflamed and inflamed conditions. In the study, cells were isolated from human dental pulp, STRO-1-immunomagnetically sorted, and seeded out to obtain single colony-forming units. After incubation of DPSCs in basic medium, it was found that DPSCs expressed TLRs 1–10 in different quantities. The inflammatory medium (interleukin-1 beta, interferon-gamma, interferon-alpha, and tumor necrosis factor-alpha) upregulated the expression of TLR2, TLR3, TLR4, TLR5, and TLR8, downregulated TLR1, TLR7, TLR9, and TLR10, and abolished TLR6.

Contrary to our expectations, no significant differences were noted between the successful and unsuccessful MSC isolation groups with respect to the type of teeth used (Table 2). Before this study, we expected that a supernumerary tooth might predict a better MSC isolation rate. In a study, Huang et al. [27] isolated DPSCs from a supernumerary tooth of a 20-year-old healthy male patient. The stem cells were capable of differentiating into adipogenic and osteogenic lineages and expressed stem cell and differentiation markers [27]. In another study, Lee et al. [28] compared DPSCs isolated from supernumerary teeth and SHEDs in 3 age- and sex-matched patients. The levels of colony-forming unit fibroblasts and the proliferation rate of supernumerary-teeth DPSCs were slightly lower than those of SHEDs [28]. However, the ability of SHED and supernumerary-teeth DPSCs to differentiate into osteogenic, adipogenic, and chondrogenic lineages was similar. Migration assay showed that both supernumerary-teeth DPSCs and SHED rapidly migrated toward wounded areas. Supernumerary-teeth DPSCs showed altered cell growth after storage for 2 years. In particular, the population doubling time of supernumerary DPSCs increased while that of SHED remained nearly unchanged. Therefore, the team concluded that both supernumerary teeth and deciduous teeth share many characteristics, such as the ability to yield highly proliferative clonogenic cells with an immunophenotype similar to that of MSCs, although they are inferior to exfoliated deciduous teeth in terms of long-term banking [28].

Similarly, the successful and unsuccessful MSC isolation groups did not differ with respect to patient age (Table 2). Little is known about the age-related changes of DPSCs, and it remains unclear whether aging and the related changes in the microenvironment are associated with DPSCs. In a study, Ma et al. [29] exposed adult rat DPSCs to juvenile rat dental pulp cell-conditioned medium (DPC-CM), and juvenile DPSCs were exposed to adult DPC-CM. The study revealed that DPSCs isolated from the juvenile donors displayed increased proliferation and decreased osteogenic differentiation ability than those isolated from adult DPSCs. Interestingly, adult DPSCs induced by juvenile DPC-CM demonstrated enhanced proliferation but decreased osteogenic differentiation ability, whereas DPSCs from juvenile donors induced by adult DPC-CM showed decreased proliferation but enhanced ability for osteogenic differentiation [29]. In another study, Mehrazarin et al. [30] revealed that dental MSCs lose their odontogenic differentiation potential during senescence, partly because of reduced Bmi-1 expression.

## 5. Conclusion

In summary, the data suggest that a carious deciduous tooth or tooth with pulpitis was relatively unsuitable for MSC processing and isolation. This observation is particularly important for dental stem cell banking and regenerative dentistry. Nevertheless, whether the stem cell can be isolated is one question but its population following the isolation still lies as the most significant question.

## Figures and Tables

**Table 1:** List of primers used in this study.

Primer	Sequence
Glyceraldehyde 3-phosphate dehydrogenase (GAPDH)	TTCCAGGAGCGAGATCCCT
Caspase-1	ABI Hs00354836_m1
Interleukin-6 (IL-6)	Hs02621719_u1
Monocyte chemoattractant protein-1 (MCP-1)	GCAATCAATGCCCCAGTCA
Toll-like receptor 1 (TLR1)	AACCCATTCCGCAGTACTCCA
Toll-like receptor 2 (TLR2)	CAATGATGCTGCCATTCTCAT
Toll-like receptor 3 (TLR3)	ACAACTTAGCACGGCTCTGGA
Toll-like receptor 4 (TLR4)	AGTTTCCTGCAATGGATCAAGG
Toll-like receptor 5 (TLR5)	GGCTTAATCACACCAATGTCACTAT
Toll-like receptor 6 (TLR6)	CCCATTCCACAGAACAGCAT
Toll-like receptor 7 (TLR7)	TGGAAATTGCCCTCGTTGTT
Toll-like receptor 8 (TLR8)	CTTCGATACCTAAACCTCTCTAGCAC
Toll-like receptor 9 (TLR9)	CTAGCTCTTAATCCTGATG

**Table 2:** Baseline characteristics of the collected deciduous dental pulps (*N* = 161).

	Total (*N* = 161)	Successful isolation of MSCs (*N* = 128)	Unsuccessful isolation of MSCs (*N* = 33)	*P*
Age, year	8.590 ± 2.782	8.631 ± 2.885	8.431 ± 2.373	0.715
Male, *n* (%)	115 (71.4)	945 (74.2)	20 (60.6)	0.123
Interval between extraction to culture, day	1.652 ± 3.469	1.914 ± 3.845	0.636 ± 0.381	0.059
*Types of teeth*				0.219
Incisor, *n* (%)	31 (19.3)	26 (20.3)	5 (15.2)	
Canine, *n* (%)	21 (13.0)	16 (12.5)	5 (15.2)	
Molar, *n* (%)	61 (37.9)	44 (34.4)	17 (51.5)	
Supernumerary, *n* (%)	48 (29.8)	42 (32.8)	6 (18.2)	
*Severity of dental caries*				<0.001^*∗∗∗*^
No, *n* (%)	86 (53.4)	81 (63.3)	5 (12.5)	
Mild-to-moderate, *n* (%)	45 (28.0)	31 (24.2)	14 (42.4)	
Severe, *n* (%)	30 (18.6)	16 (12.5)	14 (42.4)	
*Pulpitis*				<0.001^*∗∗∗*^
No, *n* (%)	139 (86.3)	122 (95.3)	17 (51.5)	
Yes, *n* (%)	22 (13.7)	6 (4.7)	16 (48.5)	

Note: MSC mesenchymal-like stem cells. ^*∗∗∗*^*P* < 0.001.

**Table 3:** Logistic regression analysis for successful isolation of MSCs derived from deciduous dental pulp (*N* = 161).

Variable	Univariate analysis			Multivariate analysis		
	OR	95% CI	*P*	OR	95% CI	*P*
Absence of dental caries	9.651	3.490–2689	<0.001^*∗∗∗*^	4.741	1.564–14.371	0.006^*∗∗*^
Absence of pulpitis	19.137	6.586–55.607	<0.001^*∗∗∗*^	9.111	2.921–28.420	<0.001^*∗∗∗*^

Note: MSC: mesenchymal-like stem cells, CI: confidence interval, and OR: odds ratio. ^*∗∗*^*P* < 0.01; ^*∗∗∗*^*P* < 0.001.
